# Kin Competition Drives the Evolution of Earlier Metamorphosis

**DOI:** 10.1002/ece3.70806

**Published:** 2025-01-14

**Authors:** Bing Dong, Andy Gardner

**Affiliations:** ^1^ School of Biology University of St Andrews Dyers Brae St Andrews UK

**Keywords:** complex life cycle, Hamilton's rule, inclusive fitness, kin competition, kin selection, metamorphosis

## Abstract

Metamorphosis, the discrete morphological change between postembryonic life stages, is widespread across the animal kingdom. The suggested advantages of metamorphosis have usually been framed in terms of population benefits, i.e., ecological explanations. In contrast, evolutionary explanations concern whether and how metamorphosis spreads through a population owing to individual‐fitness benefits. However, how kin selection modulates evolution of metamorphosis remains to be investigated formally. Here we develop a mathematical model to investigate how kin selection shapes the optimal timing of metamorphosis from foraging, non‐reproductive larva to reproductive adult, when larvae tend to cluster with their genetic relatives. We consider the full range of larval competition intensities—from no competition to full competition—and the full range of relatedness coefficients—from unrelated to clonality. We provide testable predictions as to how kin selection modulates the timing of metamorphosis, as well as a conceptual framework within which empirical observations may be understood.

## Graphical abstract

We develop a mathematical model to investigate how kin selection shapes the optimal timing of metamorphosis. We consider the full range of larval competition intensities and the full range of relatedness coefficients. This yields testable predictions as to how kin selection modulates the timing of metamorphosis.

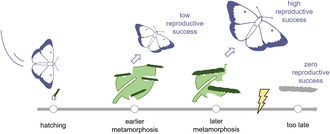

## 1 Introduction

Metamorphosis, the discrete morphological change between postembryonic life stages, is widespread across the animal kingdom (Werner 1988; Truman 2019). Adaptive explanations (Table 1) treat metamorphosis as a means of niche shift (e.g., diet and habitat) or fitness‐role shift (e.g., feeding, dispersal and reproduction) that decouples early and late life stages (Istock 1967; Ebenman 1992; Moran 1994; ten Brink, de Roos, and Dieckmann 2019). One consequence of these shifts is that competition between juveniles and adults is reduced (Istock 1967; Haefner and Edson 1984; Pechenik 1999). Competition within life stages can also be reduced as one individual's metamorphosis may free‐up resources for neighbouring individuals that are yet to undergo metamorphosis (Rowe and Ludwig 1991; Pechenik 1999).

**TABLE 1:** Major adaptive explanations for metamorphosis.

Hypothesis	Prediction	Reference
Adaptive decoupling	Metamorphosis enables juvenile and adult to evolve separately	Istock (1967); Ebenman (1992); ten Brink, de Roos, and Dieckmann (2019)
Niche shift (habitat)	Metamorphosis is adaptive for spatially heterogeneous environment	Imms (1964); Bryant (1969)
	Metamorphosis is adaptive for temporarily heterogeneous environment	Wilbur (1980); Haefner and Edson (1984)
Predation	Structure like insect wings increases predator escape	Bryant (1969)
Niche shift (diet)	Metamorphosis enables larval exploitation of temporary food	Wassersug (1975); Slade and Wassersug (1975)
	Metamorphosis enables specialisation on secondary food resource	Lameere (1899); ten Brink, de Roos, and Dieckmann (2019)
Size‐dependent growth & mortality rate	At smaller size, a larval form has higher growth or lower mortality rate	Gilliam (1982)
Nepotism	Kin recognition and limited dispersal promote co‐settlement	Grosberg and Quinn (1986)
Fitness role (habitat selection)	Short planktonic non‐feeding larval stage and early settling is favoured	Strathmann (1993)
	Adult form is adaptive for reproductive site selection	Moran (1994)
Fitness role (mating)	Wing retention in males but not females is favoured	Moran (1994)
Competition	Metamorphosis reduces intraspecific competition	André (1991); Haefner and Edson (1984)
Kin competition	Selection for spreading sibling larvae favours long pelagic larval phase	Strathmann (1974)
	Dispersive larval form of sedentary species is favoured	Moran (1994)

The suggested advantages of metamorphosis have often been framed in terms of benefits for the population or species, i.e., ecological explanations (Istock 1967; Haefner and Edson 1984). In contrast, consideration as to whether and how metamorphosis can spread through a population owing to individual‐fitness advantages yield evolutionary explanations (Moran 1994; ten Brink, de Roos, and Dieckmann 2019). Kin selection has been suggested to play a role in the evolution of a non‐feeding dispersive stage in taxa characterised by sessile adults (Strathmann 1974; Moran 1994). However, how kin selection modulates the evolution of metamorphosis remains to be investigated formally.

Here we develop a mathematical model to investigate how kin selection acts in relation to the optimal timing of metamorphosis from foraging, non‐reproductive larva to reproductive adult. We consider the full range of competition intensities—from no competition, whereby a larva's foraging success is independent of the number of neighbouring larvae, to full competition, whereby the larva's foraging success is inversely proportional to the number of neighbouring larvae—and the full range of genetic relatedness between larvae within the same patch—from unrelated to genetically identical. Our aim is to provide testable, comparative predictions concerning the effect of kin selection on the timing of metamorphosis as well as a conceptual framework within which empirical observations may be understood.

## 2 Results

**FIGURE 1:**
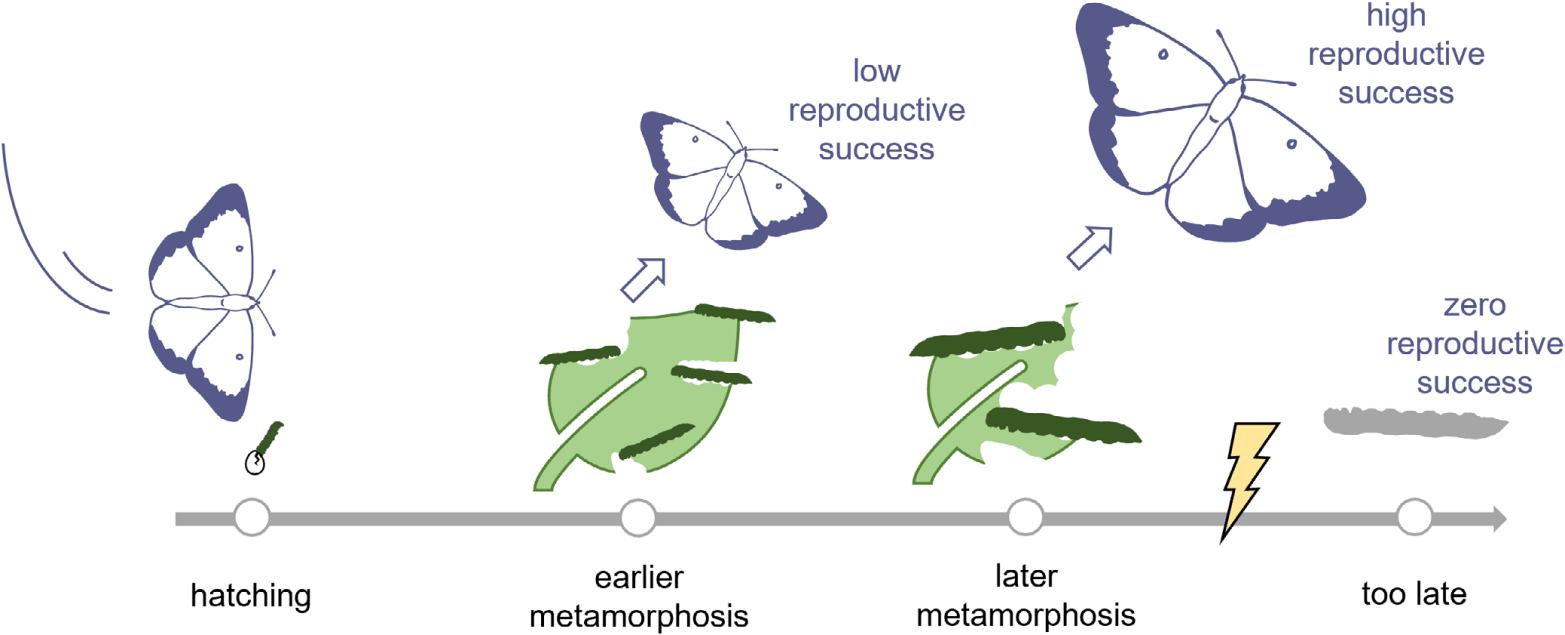
Juveniles that undergo metamorphosis earlier accrue less resource and hence enjoy only limited reproductive success as adults, whereas juveniles that undergo metamorphosis later accrue more resource and hence enjoy greater reproductive success as adults, but juveniles that do not undergo metamorphosis before a catastrophe strikes their natal patch are killed and have zero reproductive success. Moreover, to the extent that there is competition for resources, juveniles that undergo earlier metamorphosis free‐up resources for their patch mates—to whom they may be genetically related.

We consider a patch‐structured population with morphologically distinct juvenile and adult stages and non‐overlapping generations. A large number of juveniles hatch on each patch, and each juvenile accrues a food resource continuously at a rate given by a power function *ρn* ^−*α*^ of the current number of juveniles *n* on her patch, with scaling constant *ρ* being positive and the exponent parameter *α* taking a value between zero (i.e., no competition) and one (i.e., full competition). The individual accrues resources either until she undergoes metamorphosis to become an adult or else until a catastrophe destroys all juveniles currently resident in her patch. Each individual undergoes metamorphosis at a constant rate *μ* determined by her genotype, such that her waiting time until metamorphosis is an exponentially distributed random variable *t*. Upon undergoing metamorphosis to adulthood, an individual stops accruing the juvenile resource and disperses away from the patch to compete with other adults across the whole population for reproductive opportunities in the next generation of patches, with her expected reproductive success being proportional to the amount of resource she accrued as a juvenile, perhaps—for example—on account of her being better able to compete for access to breeding spots. That is, the longer she has spent accruing resources as a juvenile the more resources she has accrued and hence the greater her expected reproductive success, conditional upon her having undergone metamorphosis before a catastrophe has occurred (Figure 1). Catastrophes befall the patch at a constant rate *τ*, such that the waiting time until catastrophe is an exponentially distributed random variable *T*.

Under these assumptions, a focal individual's fitness may be expressed as

(1) $$w\left(\mu ,\nu \right)=\int_{0}^{\infty }\tau e^{-\mathrm{\tau T}}\left(\int_{0}^{T}{\mathrm{\mu e}}^{-\mathrm{\mu t}}\left(\int_{0}^{t}\rho {\left(n_{0}e^{-\mathrm{\nu x}}\right)}^{-\alpha }dx\right)dt\right)dT$$

where *ν* is the average rate of metamorphosis among the juveniles on her patch, *n*_0_ is the initial number of juveniles on the patch and the variable *x* denotes a moment of time before the focal individual undergoes metamorphosis. Here: the innermost integral describes the amount of resource accrued by the focal individual by time *t*, conditional upon her not having undergone metamorphosis before this time; the middle integral describes the expected amount of resource she accrues before a catastrophe strikes at time *T*, taking into account that she may have undergone metamorphosis before this time; and the outermost integral describes the expected amount of resource she is able to invest into reproductive opportunities as an adult (with this being zero if she does not survive to adulthood).

Applying neighbour‐modulated fitness kin‐selection methodology (Taylor 1996; Taylor and Frank 1996; Frank 1997, 1998), we find that the condition for natural selection to favour an increase in the rate at which individuals undergo metamorphosis is given by ∂*w*/∂μ + ∂*w*/∂ν × *r* > 0 or, equivalently, by

(2) $$\frac{\tau }{m\left(m+\tau \right)}-\frac{1}{m\left(1-\alpha \right)+\tau }+\frac{\alpha }{m\left(1-\alpha \right)+\tau }r>0$$

where *m* is the average rate of metamorphosis across the whole population, *r* is the kin‐selection coefficient of genetic relatedness and all derivatives are evaluated at *μ* = *ν* = *m* (see Appendix A for details). Condition (2) takes the form of Hamilton's (1963, 1964, 1970) rule, and the terms on the left‐hand side can be interpreted as the inclusive‐fitness consequences for an actor who slightly increases her rate of undergoing metamorphosis. Specifically: the first term *τ*/(*m*(*m* + *τ*)) describes the benefit of increased survival to adulthood by undergoing metamorphosis before the catastrophe occurs; the second term −1/(*m*(1 − *α*) + *τ*) describes the cost of reduced reproductive success during adulthood on account of accruing fewer resources as a larva; and the third term (*α*/(*m*(1 − *α*) + *τ*))*r* describes the kin‐selected benefit of having freed‐up resources for genetically related larval competitors. At evolutionary equilibrium, the direct‐fitness effect is negative and the indirect‐fitness (i.e., kin‐selected) effect is positive, such that an increased rate of metamorphosis is formally altruistic (Hamilton 1964) (see Appendix A for details).

Setting the left‐hand side of condition (2) equal to zero and solving for *m* obtains the optimal rate of metamorphosis, m* (see Appendix A for details). Note that the expected waiting time until metamorphosis, measured in units of the expected waiting time until catastrophe, is *M* = *τ*/*m* and, accordingly, the optimal timing of metamorphosis may be expressed as in dimensionless terms as M* = *τ*/m* or

(3) $$M^{*}=\frac{2\left(1-\mathrm{r\alpha}\right)}{\sqrt{4\left(1-\mathrm{r\alpha}\right)+{\left(1-r\right)}^{2}\alpha^{2}}-\left(1-r\right)\alpha }$$

**FIGURE 2:**
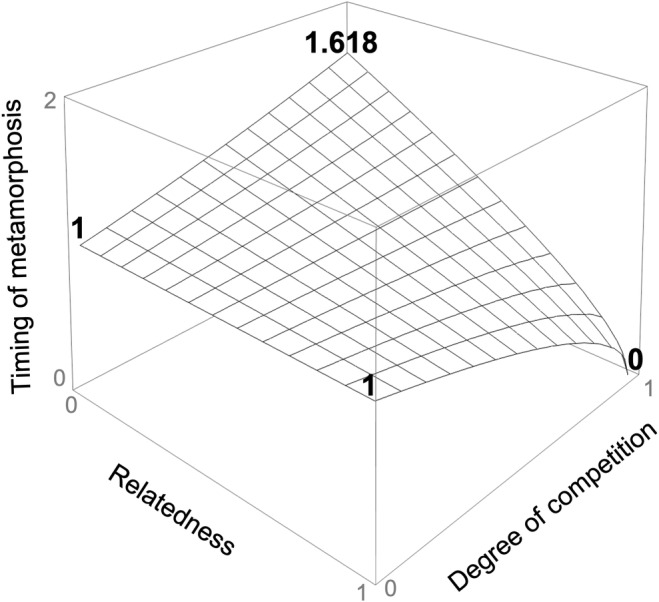
The optimal timing of metamorphosis (M*) is modulated by genetic relatedness (*r*) and degree of competition (*α*). In the absence of larval competition (*α* = 0), the optimal timing of metamorphosis is independent of the relatedness between larvae. However, when there is competition between larvae (*α* > 0), higher larval relatedness promotes earlier metamorphosis. When relatedness is low (*r* < ½), higher larval competition promotes later metamorphosis; and when relatedness is high (*r* > ½), higher larval competition promotes earlier metamorphosis. In the special case of relatedness equal to one‐half (i.e., *r* = ½, such as for full‐siblings under outbreeding and diploidy), the optimal timing of metamorphosis is independent of the degree of competition.

This reveals that earlier metamorphosis is favoured as genetic relatedness increases, so long as there is some competition for resources (i.e., dM*/d*r* ≤ 0 when *α* > 0). This is because earlier metamorphosis frees up reproductive resources for one's relatives. Moreover, later metamorphosis is favoured as resource competition increases when relatedness is less than one‐half (i.e., dM*/d*α* ≥ 0 when *r* ≤ 1/2), and earlier metamorphosis is favoured as resource competition increases when relatedness is greater than one‐half (i.e., dM*/d*α* ≤ 0 when *r* ≥ 1/2; Figure 2). This is because as the degree of resource competition increases a larva's foraging success increases more rapidly over time on account of her resource competitors diminishing in number; when her relatedness to these competitors is low then she is favoured to exploit this enhanced foraging success by delaying metamorphosis, and when her relatedness to the competitors is high she is favoured to hasten metamorphosis so as to allow them to exploit the resources that she has freed up. In the special case of no competition (i.e., *α* = 0), the optimal timing of metamorphosis is M* = 1, independently of the degree of relatedness (Figure 2): this means that only 50% of juveniles undergo metamorphosis before the catastrophe occurs. In the special case of full competition (i.e., *α* = 1), the optimal timing of metamorphosis decreases from M* = ($\sqrt{5}$ + 1)/2 ≈ 1.618 (the golden ratio) when patch mates are unrelated (i.e., *r* = 0), such that only 38% of juveniles undergo metamorphosis before the catastrophe occurs, down to M* → 0 in the limit of patch mates being clonally related (i.e., *r* → 1), such that 100% of juveniles undergo metamorphosis before the catastrophe occurs.

## 3 Discussion

We have performed a first formal analysis of the role of kin selection in the evolution of metamorphosis, with a focus on the timing of metamorphosis, and considering a range of relatedness and competition scenarios. Our aim has been to develop a relatively generic, illustrative model that captures the basic trade‐offs in a phenomenological way, rather than a more concrete model tailored to a specific setting. Our analysis shows that, when there is competition between juveniles, earlier metamorphosis is an altruistic act that increases the fitness of neighbouring juveniles by freeing‐up reproductive resources while also reducing the actor's own fitness, and accordingly higher relatedness between juveniles is predicted to be associated with earlier metamorphosis. This effect of relatedness is expected to completely disappear in the absence of resource competition between larvae. Conversely, a higher intensity of larval competition is predicted to favour later metamorphosis when relatedness is low and earlier metamorphosis when relatedness is high—with the intensity of competition having little impact on the timing of metamorphosis when relatedness is intermediate.

We have treated relatedness as a simple parameter that may take values between zero and one, which provides coverage for a wide range of particular systems. For example, we have not been required to specify whether reproduction is asexual or sexual and, in the event of the latter, we have not been required to specify details as to ploidy or whether females mate monogamously versus multiply—the model covers all these possibilities. In the case of clonal, asexual reproduction, any degree of relatedness between zero and one may be obtained by adjusting the number and/or reproductive skew of mothers contributing offspring to a single patch. In the case of sexual, diploid reproduction, the maximum relatedness among patch mates will typically be one‐half, i.e., when a single, monogamously mated mother contributes offspring to the patch, and the relatedness of patch mates may be lower than this maximum on account of female promiscuity and/or multiple mothers contributing offspring to each patch.

Our analysis has revealed that, in the special case of full competition (*a* = 1) among completely unrelated juveniles (*r* = 0), natural selection favours an expected time to metamorphosis that is ϕ ≈ 1.618 times the expected waiting time until catastrophe. This is the golden ratio, defined as the ratio of two numbers whereby the ratio of the sum of the two numbers to the larger number takes the same value; as such, it is the asymptotic ratio of consecutive terms of the Fibonacci sequence. The golden ratio and Fibonacci sequence are found throughout nature, ranging from the number of petals on some flowers, and the shape of snail shells, to the branching patterns of coronary arteries in the human body (Land 1960; Yalta, Ozturk, and Yetkin 2016). However, our golden ratio result is unlikely to be robust to changes in model assumptions, for example relaxing the assumption that adult reproductive success is exactly proportional to the amount of resource accrued as a juvenile.

In our analysis, we have assumed that an individual's rate of resource intake is independent of her timing of metamorphosis; however, it may be more reasonable to assume that individuals exhibiting a higher rate of resource uptake will also exhibit earlier metamorphosis—i.e., they consume resources faster so as to grow more quickly and hence more rapidly attain a body size that is appropriate for undergoing metamorphosis. If there is a trade‐off between feeding rate and feeding efficiency (Deng et al. 2003; Kotrschal, Szidat, and Taborsky 2014) then earlier metamorphosis may decrease the overall efficiency of resource use within a neighbourhood, such that—all else being equal—earlier metamorphosis may represent a selfish act. In that case, higher juvenile relatedness would tend to favour later metamorphosis. Moreover, we assume that metamorphosis occurs at a constant rate determined by the individual's genotype, such that relatives undergo metamorphosis at different times; more synchronous metamorphosis would tend to be less effective in reducing kin competition. Such extensions to the present model represent useful avenues for further formal analysis, to determine the overall impact of relatedness on the timing of metamorphosis.

Our analysis has focused on organisms with metamorphosis whose reproductive success depends on the amount of resource accrued as larvae. An extreme example of this is provided by taxa in which adults do not feed at all, such as in many orders of holometabolous insects, including all mayflies (Ephemeroptera; Rowe and Ludwig 1991), some moths (e.g., Saturniidae; Tuskes, Collins, and Tuttle 1996), and some fireflies (e.g., *Lampyris noctiluca* ; Baudry et al. 2021). For taxa with a dispersive and non‐feeding larval stage, such as many marine invertebrates (e.g., barnacle *Balanus glandula* in Hentschel and Emlet 2000; and copepods in Twombly 1996), reproductive success depends on how much resource the adult accumulates, and if adults are somewhat related to neighbouring individuals (e.g., acorn barnacles *Semibalanus balanoides* , see Veliz et al. 2006), then later metamorphosis may mean further dispersal and hence lower relatedness between resource competitors, such that delayed metamorphosis may be favoured as a means of alleviating kin competition.

Alternatively, individuals might also enjoy benefits from associating with kin—as appears to be the case in the ascidian *Botryllus schlosseri* , in which histocompatible neighbours more readily fuse to form colonies (Grosberg and Quinn 1986)—and in such cases it is feasible that kin selection may favour reduced dispersal and hence an earlier metamorphosis. More generally, cooperation among kin might facilitate the evolution of more extreme metamorphosis insofar as larvae that enjoy parental and/or alloparental care are able to focus on feeding and growing (Hölldobler and Wilson 1990), with other individuals taking care of foraging and their safety. Just as the presence of workers in a social insect nest relaxes constraints on the morphological adaptations of their queen, enabling her to focus her effort on reproduction (Hölldobler and Wilson 1990), so too might the cared‐for larva be able to evolve into little more than a specialised digestive tract, making the transformation to fully functional adult more morphologically striking. These possibilities represent useful avenues for future theoretical investigation.

## Author Contributions

**Bing Dong:** conceptualization (equal), formal analysis (lead), investigation (lead), methodology (equal), project administration (equal), software (lead), validation (equal), visualization (lead), writing – original draft (lead), writing – review and editing (supporting). **Andy Gardner:** conceptualization (equal), formal analysis (supporting), funding acquisition (lead), investigation (supporting), methodology (equal), project administration (equal), software (supporting), supervision (lead), validation (equal), visualization (supporting), writing – original draft (supporting), writing – review and editing (lead).

## Conflicts of Interest

The authors declare no conflicts of interest.

## Data Availability

All the data (equations and derivations) are included in this manuscript, in Results or Appendix A.

## Appendix A

### A.1 Derivation of Optimal Timing of Metamorphosis

The focal individual's fitness, expressed relative to the population average, is *W*(*μ*, *ν*, *m*) = *w*(*μ*, *ν*)/*w*(*m*, *m*). We consider a locus G that controls the rate of metamorphosis, and denote the genic value of a gene drawn from the locus from a focal juvenile by *g*, the additive genetic breeding value (Price 1970; Falconer 1981) for the rate of metamorphosis of the focal individual's mother by $\tilde{g}$, the average genetic breeding value of a random adult female on same patch by ${\tilde{g}}^{\prime }$, and the average genetic breeding value of the population by $\bar{g}$. Natural selection increases average breeding value if d*W*/d*g* > 0, and this can be written as

(A1)
$$\frac{dW}{dg}=\frac{\partial W}{\partial \mu }\frac{\mathrm{d\mu}}{d\tilde{g}}\frac{d\tilde{g}}{dg}+\frac{\partial W}{\partial \nu }\frac{\mathrm{d\nu}}{d{\tilde{g}}^{\prime }}\frac{d{\tilde{g}}^{\prime }}{dg}=\left(\frac{\partial W}{\partial \mu }p_{I}+\frac{\partial W}{\partial \nu }p_{S}\right)\gamma >0$$

where *p*_I_ is the coefficient of consanguinity of an individual to herself, *p*_s_ is the coefficient of consanguinity (Bulmer 1994) of an individual to a random social partner at the same patch, *γ =* d*μ/*d $\tilde{g}$ = d*ν*/d ${\tilde{g}}^{\prime }$ is the mapping between genotype and phenotype (which is the same for all individuals); and all derivatives are evaluated at the population average (*g* = $\bar{g}$). According to Hamilton's (1963, 1964, 1970) rule−*c* + *b r* > 0, ∂*W/*∂*μ* = −*c* can be interpreted as the cost incurred by increasing the rate of metamorphosis to the individual (direct fitness), ∂*W/*∂*ν* = *b* is the benefits to social partners (indirect fitness), and the ratio *p*_s_/*p*_I_ = *r* is the kin‐selection coefficient of genetic relatedness. Making the substitution *μ* = *ν* = *m* into the condition (∂*W/*∂*μ* + ∂*W/*∂*ν r*)*γ* > 0 yields expression (2) of the main text.

Specifically, ∂*W/*∂*μ* = *τ*/*m*(*m + τ*) − 1/(*m*(1—*α*) + *τ*) and ∂*W/*∂*ν* = *α*/(*m*(1 − *α*) *+ τ*). At equilibrium, we have ∂*W/*∂*μ* + ∂*W/*∂*ν r* = 0, such that ∂*W/*∂*μ* < 0 and ∂*W/*∂*ν* > 0, which means increasing the rate of metamorphosis (higher *m*) is costly to individual herself and beneficial to her social partners, and hence it is an altruistic behaviour (Hamilton 1964; West, Griffin, and Gardner 2007).

Setting the left‐hand side of expression (2) to zero and solving for *m* = *m** yields

(A2)
$$m^{*}=\frac{\sqrt{4\left(1-\mathrm{r\alpha}\right)+{\left(1-r\right)}^{2}\alpha^{2}}-\left(1-r\right)\alpha }{2\left(1-\mathrm{r\alpha}\right)}\tau$$

As the left‐hand side of condition (2) is a decreasing function of *m*, the equilibrium rate of metamorphosis is globally convergence stable (Christiansen 1991; Taylor 1996; Taylor and Frank 1996). In the main text we refer to this convergence stable strategy *m** as the ‘optimal rate of metamorphosis’ and its reciprocal (scaled so as to be dimensionless) *M** = τ/*m** as the ‘optimal timing of metamorphosis’. Note that, when behaving optimally, the proportion of individuals that undergo metamorphosis before a catastrophe befalls their patch is

(A3)
$$P=\int_{0}^{\infty }e^{-T}\left(1-e^{-\frac{T}{M^{*}}}\right)dT=\frac{1}{1+M^{*}}$$

For example, this yields *p* = 0.38 for *M** = 1.618, *p* = 1/2 for *M** = 1 and *P* → 1 for *M** → 0, as reported in the main text.
